# Treatment outcomes 24 months after initiating short, all-oral bedaquiline-containing or injectable-containing rifampicin-resistant tuberculosis treatment regimens in South Africa: a retrospective cohort study

**DOI:** 10.1016/S1473-3099(21)00811-2

**Published:** 2022-07

**Authors:** Norbert Ndjeka, Jonathon R Campbell, Graeme Meintjes, Gary Maartens, H Simon Schaaf, Jennifer Hughes, Xavier Padanilam, Anja Reuter, Rodolfo Romero, Farzana Ismail, Martin Enwerem, Hannetjie Ferreira, Francesca Conradie, Kogieleum Naidoo, Dick Menzies

**Affiliations:** a National Department of Health, Tuberculosis Control and Management Cluster, Pretoria, South Africa; b Nelson R Mandela School of Medicine, University of KwaZulu Natal, Durban, South Africa; c Department of Epidemiology, Biostatistics, and Occupational Health and the McGill International TB Centre, McGill University, and The Montreal Chest Institute, McGill University Health Centre, Montreal, QC, Canada; d Department of Medicine and Wellcome Centre for Infectious Diseases Research in Africa, Institute of Infectious Disease and Molecular Medicine, University of Cape Town, South Africa; e Desmond Tutu TB Centre, Department of Paediatrics and Child Health, Faculty of Medicine and Health Sciences, Stellenbosch University, Cape Town, South Africa; f Sizwe Tropical Disease Hospital, Department of Health, Johannesburg, South Africa; g Medicines Sans Frontieres, Khayelitsha, South Africa; h Clinical head, District Clinical Specialist Team, Namakwa, South Africa; i Centre for Tuberculosis, National Institute for Communicable Diseases, Johannesburg, South Africa; j Department of Medical Microbiology, University of Pretoria, Pretoria, South Africa; k Amity Health Consortium, Johannesburg, South Africa; l Klerksdorp and Tshepong Hospital Complex MDR/XDR TB Unit, Northwest Provincial Department of Health, Mahikeng, South Africa; m Department of Clinical Medicine, Faculty of Health Sciences, University of Witwatersrand, Johannesburg, South Africa; n Centre for the AIDS Programme of Research in South Africa (CAPRISA), Durban, South Africa; o South African Medical Research Council (SAMRC)-CAPRISA TB-HIV Pathogenesis and Treatment Research Unit, Durban, South Africa

## Abstract

**Background:**

There is a need for short and safe all-oral treatment of rifampicin-resistant tuberculosis. We compared outcomes up to 24 months after treatment initiation for patients with rifampicin-resistant tuberculosis in South Africa treated with a short, all-oral bedaquiline-containing regimen (bedaquiline group), or a short, injectable-containing regimen (injectable group).

**Methods:**

Patients with rifampicin-resistant tuberculosis, aged 18 years or older, eligible for a short regimen starting treatment between Jan 1 and Dec 31, 2017, with a bedaquiline-containing or WHO recommended injectable-containing treatment regimen of 9–12 months, registered in the drug-resistant tuberculosis database (EDRWeb), and with known age, sex, HIV status, and national identification number were eligible for study inclusion; patients receiving linezolid, carbapenems, terizidone or cycloserine, delamanid, or para-aminosalicylic acid were excluded. Bedaquiline was given at a dose of 400 mg once daily for two weeks followed by 200 mg three times a week for 22 weeks. To compare regimens, patients were exactly matched on HIV and ART status, previous tuberculosis treatment history, and baseline acid-fast bacilli smear and culture result, while propensity score matched on age, sex, province of treatment, and isoniazid-susceptibility status. We did binomial linear regression to estimate adjusted risk differences (aRD) and 95% CIs for 24-month outcomes, which included: treatment success (ie, cure or treatment completion without evidence of recurrence) versus all other outcomes, survival versus death, disease free survival versus survival with treatment failure or recurrence, and loss to follow-up versus all other outcomes.

**Findings:**

Overall, 1387 (14%) of 10152 patients with rifampicin-resistant tuberculosis treated during 2017 met inclusion criteria; 688 in the bedaquiline group and 699 in the injectable group. Four patients (1%) had treatment failure or recurrence, 44 (6%) were lost to follow-up, and 162 (24%) died in the bedaquiline group, compared with 17 (2%), 87 (12%), and 199 (28%), respectively, in the injectable group. In adjusted analyses, treatment success was 14% (95% CI 8–20) higher in the bedaquiline group than in the injectable group (70% *vs* 57%); loss to follow-up was 4% (1–8) lower in the bedaquiline group (6% *vs* 12%); and disease-free survival was 2% (0–5) higher in the bedaquiline group (99% *vs* 97%). The bedaquiline group had 8% (4–11) lower risk of mortality during treatment (17·0% *vs* 22·4%), but there was no difference in mortality post-treatment.

**Interpretation:**

Patients in the bedaquiline group experienced significantly higher rates of treatment success at 24 months. This finding supports the use of short bedaquiline-containing regimens in eligible patients.

**Funding:**

WHO Global TB Programme.

**Translation:**

For the French translation of the abstract see Supplementary Materials section.

## Introduction

Tuberculosis is a public health emergency, resulting in around 1·5 million deaths annually.^1^ HIV co-infection and *Mycobacterium tuberculosis* strains resistant to rifampicin—the most effective tuberculosis drug—reduce the probability of treatment success. South Africa, like many other African countries, has a high burden of HIV, tuberculosis, and rifampicin-resistant tuberculosis.^1^ Approximately two-thirds of patients with rifampicin-resistant tuberculosis are people living with HIV (PLHIV). For many years, rifampicin-resistant tuberculosis treatment regimens were toxic and had poor effectiveness, leading to successful treatment outcomes in less than 50% of patients. Until 2017, the duration of treatment of rifampicin-resistant tuberculosis in South Africa was 18–24 months, including 6–8 months of an injectable agent, such as kanamycin, which resulted in ototoxicity and nephrotoxicity in many patients.

**Research in context**

**Evidence before this study**

In 2017, 57% of patients worldwide treated for rifampicin-resistant tuberculosis had a successful outcome (cure or complete treatment). Early discontinuation of therapy based on patient's decision (loss to follow-up) was a common reason for poor outcomes; loss to follow-up is associated with 18–24-month treatment duration and medication toxicity. In 2013, WHO first recommended use of a standard shorter regimen based on observational studies in Bangladesh. In 2017, this regimen was introduced in South Africa. However, high levels of ototoxicity were noted shortly after introduction. Based on South Africa's successful experience replacing these injectables with the new oral drug bedaquiline in longer rifampicin-resistant tuberculosis regimens, South Africa began also replacing the injectables used in short regimens with bedaquiline under programmatic conditions, and slowly expanded its implementation. We searched PubMed on Nov 15, 2021, using the terms ((((tuberculosis) AND ((rifampin-resistant) OR (rifampicin-resistant))) AND (all-oral)) AND (bedaquiline)) AND (short) without language restrictions. We identified only one study evaluating a bedaquiline-containing all-oral short regimen for rifampicin-resistant tuberculosis, although the regimen differed from the one we evaluated. Despite being a non-comparative study and including only three health facilities in South Africa, the study found a high treatment success rate with the bedaquiline-containing regimen.

**Added value of this study**

In this study used to inform WHO rifampicin-resistant tuberculosis treatment guidelines, we report on the outcomes from the introduction of the bedaquiline-containing short regimen in South Africa. We showed that all-oral bedaquiline-containing short regimens were associated with an absolute improvement in rifampicin-resistant tuberculosis treatment success of 14% when compared with injectable-containing short regimens. Patients receiving the bedaquiline-containing regimen had risks of mortality during treatment that were 8% lower than patients receiving injectable-containing regimen. Disease recurrence after treatment success was rare and post-treatment mortality was similar between groups. These results were robust in several subgroup analyses.

**Implications of all the available evidence**

Results from our large programmatic cohort in a high HIV-burden country provide evidence that bedaquiline used in a shorter rifampicin-resistant tuberculosis treatment regimen is associated with higher rates of treatment success 24 months after treatment initiation and associated with lower risk of mortality during treatment than an injectable-containing regimen. These results led to the 2020 WHO recommendation for the use of bedaquiline in shorter rifampicin-resistant tuberculosis regimens under programmatic conditions. Despite a short duration of treatment, disease recurrence, as measured by re-initiation of rifampicin-resistant tuberculosis treatment, was rare. Although injectable-containing short regimens had substantially lower loss to follow-up than longer regimens in previous studies, we found bedaquiline-containing short regimens might be associated with even further reductions. Clinicians and policy makers in countries that are still using injectable-based regimens might use our analysis to re-evaluate their practices.

South Africa implemented the bedaquiline clinical access programme (BCAP)^2^ from March 2013, to March 2015, to add bedaquiline for treatments of 18 months or longer. The BCAP enrolled selected patients with rifampicin-resistant tuberculosis including those with extensively drug-resistant tuberculosis—at the time defined as patients infected by *Mycobacterium tuberculosis* strains that are resistant to rifampicin, an injectable, and a fluoroquinolone.^3^ The programme resulted in substantially improved treatment outcomes with 73% of patients successfully treated.^4^ From 2015, bedaquiline was given to all patients receiving long rifampicin-resistant tuberculosis regimens who presented with ototoxicity.

A shorter, injectable-containing, WHO-recommended rifampicin-resistant tuberculosis regimen was introduced in South Africa in 2017.^3^, ^5^ This regimen, comprising kanamycin, moxifloxacin, clofazimine, ethionamide, high-dose isoniazid, ethambutol, and pyrazinamide, was given for 9–12 months. Patients with newly diagnosed rifampicin-resistant tuberculosis with no previous history of treatment with second-line anti-tuberculosis drugs and whose isolates were susceptible to injectables and fluoroquinolones were eligible to receive the regimen. However, toxicity remained a concern.^6^ Initially, in South Africa, patients initiating treatment with kanamycin received bedaquiline if kanamycin was stopped due to toxicity.^7^ However, high rates of new or worsening ototoxicity and nephrotoxicity plus poor patient acceptance related to painful injections^6^ led to a decision to instead initiate patients on short, all-oral, bedaquiline-containing rifampicin-resistant tuberculosis treatment regimen. This regimen was identical to the injectable-containing regimen, except that bedaquiline replaced kanamycin from the outset of treatment.

The objective of this study was to compare 24-month outcomes between patients initiated on an injectable-containing or bedaquiline-containing short regimen for rifampicin-resistant tuberculosis in South Africa in 2017.

## Methods

### Study design and participants

Management of rifampicin-resistant tuberculosis in South Africa is decentralised and handled at the subdistrict level across 658 facilities. All individuals with presumptive tuberculosis are tested for tuberculosis and rifampicin-resistance using the Xpert MTB/RIF assay (Cepheid, Sunnyvale, CA, USA). South Africa implemented the Xpert MTB/RIF Ultra in October 2017 in a phased approach. Guidelines state all rifampicin-resistant patients should be tested for isoniazid, rifampicin, fluoroquinolone, and second-line injectable drug resistance using GenoType MTBDR*plus* and GenoType MTBDR*sl* (Hain Lifescience, Nehren, Germany). Stable patients in fair to good clinical condition initiate rifampicin-resistant tuberculosis treatment as outpatients, whereas ill or unstable patients in poorer condition are hospitalised and initiate rifampicin-resistant tuberculosis treatment as inpatients.^8^, ^9^ All patients with rifampicin-resistant tuberculosis receive community-based or facility-based directly observed (ie, therapy observed by a health worker, family member, or treatment support worker) therapy. EDRWeb—the national electronic registry—contains all records of people initiating drug-resistant tuberculosis treatment and contains data regarding previous treatment history, patient demographics, results of drug susceptibility testing, treatment received, microbiological testing, and end of treatment outcomes from Jan 1, 2009, onwards.^10^

Records of all patients initiating treatment for rifampicin-resistant tuberculosis with or without isoniazid resistance from Jan 1 to Dec 31, 2017 using a short treatment regimen with an intended duration of less than 12 months registered in the national drug-resistant tuberculosis database EDRWeb were eligible for inclusion. In South Africa, to be eligible for either short regimen, patients had to be newly diagnosed with rifampicin-resistant tuberculosis, without history of previous treatment with second-line tuberculosis drugs, and without confirmed or suspected resistance to fluoroquinolones, second-line injectables, or both. Additional inclusion criteria for the analysis were: treatment duration of 13 months or less and receipt of the injectable-containing or bedaquiline-containing regimen. Exclusion criteria were use of cycloserine or terizidone, para-aminosalicylic acid, delamanid, carbapenems, or linezolid, and patients with missing information on the regimen they received. Additionally, we excluded patients who were aged younger than 18 years old, or had unknown HIV status, sex, or age, or their national ID number was not recorded in EDRWeb. Patients who initially received an injectable and later bedaquiline (or vice-versa) were also excluded.

This analysis was approved by Human Research Ethics Committee, Medical of University of Witwatersrand (Johannesburg, South Africa; #M210979). The requirement to obtain informed consent for individual patients was waived since this was a retrospective record review. No patients were contacted directly, nor underwent any study specific procedures.

### Procedures

The short, WHO-recommended, injectable-containing regimen consisted of 9–12 months of moxifloxacin (daily, oral 10–15 mg/kg, maximum of 400 mg) or levofloxacin (daily, oral 15–20 mg/kg, maximum of 1000 mg), clofazimine (daily, oral 2–5 mg/kg, maximum of 100 mg), ethambutol (daily, oral 15–25 mg/kg, maximum of 1200 mg), and pyrazinamide (daily, oral 20–30 mg/kg, maximum of 2000 mg), supplemented with high-dose isoniazid (daily, oral 10–15 mg/kg, maximum of 600 mg), ethionamide or prothionamide (daily, oral 15–20 mg/kg, maximum of 750mg), and an injectable (kanamycin [daily, 15–20 mg/kg, maximum of 1000 mg], amikacin [daily, 15-20 mg/kg, imaximum of 1000 mg], or capreomycin [daily, 15–20 mg/kg, maximum of 1000 mg]), delivered intramuscularly, for the first 4–6 months.^3^ The short bedaquiline-containing regimen consisted of 9–12 months of levofloxacin or moxifloxacin, clofazimine, ethambutol, and pyrazinamide (same doses and routes as the injectable-containing regimen), and supplemented with bedaquiline for the first 6 months and ethionamide or prothionamide and high-dose isoniazid (same doses and routes as the injectable-containing regimen) for the first 4 months. Bedaquiline was given at a dose of 400 mg once daily for two weeks followed by 200 mg three times a week for 22 weeks.^7^ Among patients receiving bedaquiline, levofloxacin was favoured over moxifloxacin because it has less of an effect on the QT interval.^11^

End-of-treatment outcomes were obtained from EDRWeb. End-of-treatment outcomes were recorded by the treating clinician following South Africa guidelines. To obtain information on death up to 24 months after initiation of treatment, the national ID numbers recorded in EDRWeb were matched with national death registry data.

Tuberculosis smear microscopy and culture was conducted routinely on sputum samples collected from all patients at treatment start, and monthly while on treatment thereafter. Laboratory tests, including electrolytes, renal function, liver function, thyroid function test, and full blood count, were done on the same schedule. High-frequency-enabled audiometers were used to monitor auditory function in patients receiving injectable rifampicin-resistant tuberculosis drugs. As per WHO recommendations^12^ and RSA guidelines,^7^, ^13^ Fridericia-formula corrected QT (QTcF) intervals were measured at baseline, two times in the first month and then monthly while on bedaquiline. Patients with a baseline QTcF interval of more than 450 ms, clinically significant ECG abnormality at screening, or a family history of prolonged QT syndrome were excluded from receiving bedaquiline as per treatment guidelines.

### Outcomes

End-of treatment outcomes included cure, complete treatment, treatment failure, loss to follow-up during treatment, and death during treatment.^7^

Recurrence of tuberculosis among people whose end-of-treatment outcome was recorded as complete treatment or cure was ascertained by probabilistic matching using name, date of birth, and national ID number in EDRWeb to determine if the same person was reinitiated on rifampicin-resistant tuberculosis therapy within 24 months of initiating treatment.

In this study, we defined treatment success as an end-of-treatment outcome of cure or complete treatment without evidence of death or recurrence up to 24 months after treatment initiation; failure or recurrence was defined as survival at least 24 months after treatment initiation and an end-of-treatment outcome of failure (defined according to WHO and RSA guidelines; ie, treatment terminated or discontinuation of at least two drugs due to intolerance or adverse event or drug resistance, or failure to culture convert or culture reversion after conversion) or an end-of-treatment outcome of success with evidence of recurrence during follow-up after treatment completion; loss to follow-up was defined as an end of treatment outcome of loss to follow-up (ie, treatment interruption of at least 2 consecutive months) without evidence of death; and death was defined as death of any cause up to 24 months after treatment initiation.

We retrospectively extracted the data for the patient cohort from EDRWeb on June 30, 2019, and collected 24-month outcomes for all included patients on July 31, 2020.

### Statistical analysis

The primary analysis compared 24-month outcomes among patients receiving the injectable-containing regimen (injectable group) with those receiving the bedaquiline-containing regimen (bedaquiline group). We considered multiple outcome comparisons between the regimens: success versus all other outcomes; survival versus death; disease-free survival versus survival with treatment failure or recurrence (excluding patients who died); and loss to follow-up versus all other outcomes.

To compare outcomes between treatment groups, we did binomial linear regression. We used a combination of exact and propensity-score based matching to generate similar patient populations and minimise confounding. To assess which variables to exactly match and which variables to propensity-score match for multivariable analysis, we conducted univariable analysis on a-priori selected covariates for the comparison of success versus all other outcomes. This comparison was chosen because it encompasses the most important comparison used in country-level and WHO-level measures of effectiveness of rifampicin-resistant tuberculosis treatment. These variables included age, sex, HIV (with or without antiretroviral therapy [ART]), previous treatment with first-line drugs, isoniazid resistance, acid fast bacilli (AFB) smear positivity 4 weeks before or 2 weeks after treatment start, and culture positivity 4 weeks before or 2 weeks after treatment start. Using this analysis, we exactly matched patients on HIV (negative, positive on ART, or positive without ART), AFB smear (positive, negative, or unknown), culture (positive, negative, or unknown), and previous treatment with first-line drugs (yes or no), whereas propensity-score matched using a caliper distance of 0·5 SD^14^ of the logit propensity score for age (continuous), sex (man or woman), isoniazid resistance (susceptible, resistant, or unknown), and province where patient received treatment (any of the nine provinces in South Africa) in multivariable analyses. Matching was done without replacement in a 1:1 ratio. We visually assessed the balance of matching covariates using Love plots.^15^ Through multivariable analyses we estimated the adjusted risk differences (aRD) and corresponding 95% CIs for each of our outcome comparisons between treatment regimens. In stratified analysis, we repeated these analyses for different populations: PLHIV receiving ART, HIV-negative patients, patients AFB smear-positive at baseline, patients AFB smear-negative at baseline, patients previously treated with first-line tuberculosis drugs, and patients never previously treated for tuberculosis. We conducted a post-hoc sensitivity analysis using a stricter caliper distance of 0·2 SDs during propensity score matching to see if this might affect the findings of our primary analysis.

In secondary analysis, we first constructed curves for the cumulative incidence of death from treatment initiation to 24 months between the injectable and bedaquiline group on the matched population from our primary analysis of survival versus death. Next, using the entire population, we performed an analysis of factors associated with death at any time, death during treatment, and death post-treatment. In this analysis, we did not use matching as we were interested in the potential role of different risk factors. We did multivariable binomial linear regression on the total population (ie, both treatment groups with a covariate for regimen received) and on the population stratified by received treatment; we included the same variables in the multivariable model as we did in the matched analysis. As we noticed the strongest effect on mortality was early during treatment, we conducted a post-hoc sensitivity analysis evaluating factors for early death (death within 3 months of treatment initiation) and late death (death at least 3 months after treatment initiation). Finally, we estimated median time to death from treatment initiation in the bedaquiline and injectable groups and compared them using a Kruskal-Wallis test.

All data analysis was performed in R (version 4.0.3), with matching done using the package MatchIt (version 4.1.0).

### Role of the funding source

The sponsor of the study had no role in study design, data collection, data analysis, data interpretation, or writing of the report.

## Results

**Figure 1:**
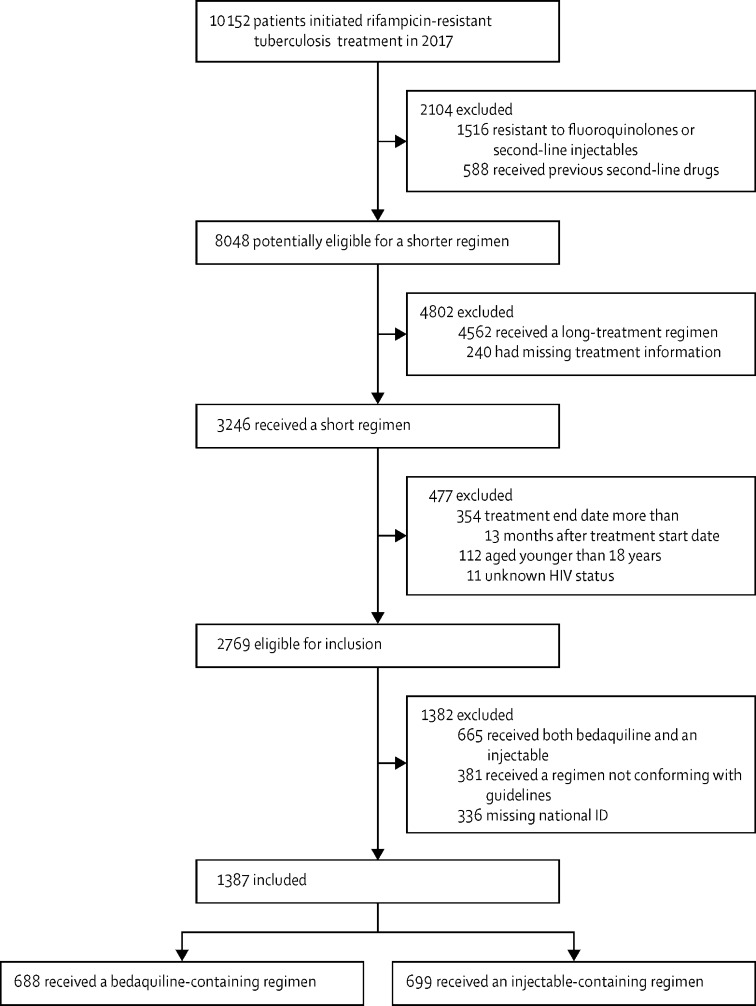
Study profile

At the time of data extraction, 10 152 patients were initiated on rifampicin-resistant tuberculosis treatment in South Africa during 2017. Among these patients, 1516 (15%) had resistance to a fluoroquinolone, second-line injectable, or both, and 588 (6%) had received previous treatment with second-line drugs, making them ineligible for short regimens. Of the remaining 8048 patients, 5279 (66%) were excluded, most commonly for receiving a longer (ie, intended duration of at least 18 months) regimen (n=4,562). 3246 patients had received a short regimen, but 477 were excluded, mainly because the treatment end date was more than 13 months after treatment starting date (n=354). Of the 2769 patients eligible for inclusion, 1382 patients were excluded, most commonly due to receiving both bedaquiline and an injectable drug (n=665). Overall, 1387 patients were included in this analysis, of whom 688 were in the bedaquiline group and 699 were in the injectable group (figure 1). The distribution of included patients by province of treatment is shown in the appendix 2 (p 2).

Patients in the bedaquiline group were older (table 1), with a median age of 42 years (IQR 33–51) versus 34 years (28–42; p<0·0001) in the injectable group. Patients in the bedaquiline group were also more likely to be men (p=0·032). There were similar proportions of PLHIV in both groups (p=0·13). Although PLHIV in the bedaquiline group were more likely to have received ART (p=0·0054), ART use in both groups was greater than 90%. Among patients in the injectable group, kanamycin was the most common injectable (595 [85%] of 699 patients); three individuals received two different injectables during treatment.

**Table 1:** Patients' demographic and clinical characteristics

		**Bedaquiline group (n=688)**	**Injectable group (n=699)**	**p value**
**Patient characteristics**				
Median age		42 (33–51)	34 (28–42)	<0·0001
Sex				
	Male	423 (61%)	389 (56%)	0·032
	Female	265 (39%)	310 (44%)	..
PLHIV		493 (72%)	474 (68%)	0·13
On antiretroviral therapy		478/493 (97%)	440/474 (93%)	0·0054
Microbiological findings at baseline*				
AFB smear positive		297/604 (49%)	248/570 (44%)	0·059
Culture positive		342/470 (73%)	334/460 (73%)	1·00
Culture, AFB, or Xpert positive		602/661 (91%)	605/663 (91%)	0·99
**Previous treatment**				
Previously treated for drug susceptible tuberculosis†		274 (40%)	286 (41%)	0·72
**Treatment received**				
Moxifloxacin or Levofloxacin		688 (100%)	699 (100%)	..
Bedaquiline		688 (100%)	0 (0%)	..
Clofazimine		688 (100%)	699 (100%)	..
Injectable		..	699 (100%)	..
Amikacin		..	106 (15%)	..
Kanamycin		..	595 (85%)	..
Capreomycin		..	1 (<1%)	..
Streptomycin		..	0	..
Mean number of other drugs received (SD)		3·0 (0·2)	2·9 (0·2)	..
**End-of-treatment outcomes**				
Treatment success		507 (74%)	421 (60%)	..
Failure		5 (1%)	14 (2%)	..
Died during treatment		117 (17%)	159 (23%)	..
Lost to follow-up		59 (9%)	105 (15%)	..
**Post-treatment outcomes**				
Returned for treatment (recurrence after success)		1 (<1%)	4 (1%)	..
Post-treatment deaths		45 (7%)	40 (6%)	..
**Final treatment outcomes 24 months post-initiation**‡				
Treatment success		478 (69%)	396 (57%)	..
Treatment failure and recurrence		4 (1%)	17 (2%)	..
Died		162 (24%)	199 (28%)	..
Lost to follow-up		44 (6%)	87 (12%)	..

Data are median (IQR), n (%), n/N (%), or mean (SD). AFB=acid fast bacilli. PLHIV=people living with HIV.

* Percentages calculated based on the number of patients with a result at baseline (baseline refers to 4 weeks before or 2 weeks after treatment initiation).

† Among patients in the bedaquiline group, 209 were treated for recurrence, 42 previous treatment failures, 21 retreatments due to loss to follow-up, and two unknown reasons; among patients in the injectable group, 201 were recurrences, 43 previous treatment failures, 36 retreatments due to loss to follow-up, and six unknown reasons.

‡ Events are classified as following: all people dying in the post-treatment follow-up period up to 24 months after treatment initiation were reclassified as deaths, regardless of other post-treatment outcomes (ie, recurrence).

The characteristics of patients eligible for inclusion in the study who were ultimately included versus those excluded are shown in appendix 2 (p 3). Included patients were older and less likely to have HIV infection or be culture positive at baseline; other characteristics were similar between included and excluded patients.

Among patients included in the analysis, 661 (96%) of 688 patients in the bedaquiline group and 663 (96%) of 699 patients in the injectable group had a valid culture, smear, or Xpert result in the baseline period. Of patients with a valid result, 602 (91%) of 661 patients in the bedaquiline group and 605 (91%) of 663 patients in the injectable group had at least one of these tests positive.

At the end of rifampicin-resistant tuberculosis treatment, 507 (74%) of 688 patients in the bedaquiline group and 421 (60%) of 699 in the injectable group were considered to have treatment success. After successful treatment and up to 24 months from treatment initiation, one (<1%) of 507 patients experienced recurrence in the bedaquiline group compared with four (1%) of 421 patients in the injectable group. Four patients (1%) had treatment failure or recurrence, 44 (6%) were lost to follow-up, and 162 (24%) died in the bedaquiline group, compared with 17 (2%), 87 (12%), and 199 (28%), respectively, in the injectable group.

Univariable analysis of different covariates showed that HIV, previous treatment, baseline AFB smear positivity, and baseline culture were most strongly associated with 24-month outcomes (appendix 2 p 4) and thus were exactly matched on in multivariable analysis.

In multivariable analysis, the matching procedure reduced the standardised mean difference between groups for all variables (appendix 2 p 5), retaining 66–70% of patients in analyses of the total population and 57–75% of patients in subgroup analyses. We found that those in the bedaquiline group had absolute rates of treatment success that were 14% (95% CI 8–20) higher than those in the injectable group (table 2). This effect remained consistent between the groups in comparisons of survival (aRD 8%; 95% CI 3–14), and disease-free survival (2%; 0–5). Additionally, patients in the bedaquiline group had lower risk of loss to follow-up during treatment compared with patients in the injectable group (aRD –4%; –8 to –1). Among all subgroups analysed, the bedaquiline group had significantly higher probability of treatment success (table 2). In a post-hoc sensitivity analysis using a caliper distance of 0·2 SDs, the standardised mean difference between groups was further reduced (appendix 2 p 6), and 44–63% of patients were retained across all analyses. Results were qualitatively identical to our primary analysis for the total population and stratified populations, with patients in the bedaquiline group having significantly higher probability of treatment success (appendix 2 p 7).

**Table 2:** Primary analysis outcomes calculated with multivariable analysis using caliper distance of 0·5 SDs for propensity score

	**Bedaquiline group (events/total)**	**Injectable group (events/total)**	**Matched pairs (% of patients matched)**	**Adjusted risk difference, % (95% CI)**
**Total population**				
Success versus all other outcomes	478/688	396/699	485 (70%)	14% (8 to 20)
Survival versus death	526/688	500/699	485 (70%)	8% (3 to 14)
Disease-free survival versus survival with treatment failure or recurrence	522/526	483/500	338 (66%)	2% (0 to 5)
Loss to follow-up versus all other outcomes	478/688	396/699	485 (70%)	−4% (−8 to −1)
**PLHIV receiving antiretroviral therapy**				
Success versus all other outcomes	337/478	245/440	345 (75%)	14% (7 to 22)
Survival versus death	372/478	302/440	345 (75%)	10% (3 to 16)
Disease-free survival versus survival with treatment failure or recurrence	368/372	296/302	238 (71%)	1% (−1 to 3)
Loss to follow-up versus all other outcomes	31/478	51/440	345 (75%)	−5% (−9 to −1)
**People HIV-Negative**				
Success versus all other outcomes	138/195	137/225	128 (61%)	16% (4 to 27)
Survival versus death	147/195	179/225	128 (61%)	4% (−7 to 14)
Disease-free survival versus survival with treatment failure or recurrence	147/147	169/179	93 (57%)	4% (0 to 8)
Loss to follow-up versus all other outcomes	9/195	32/225	128 (61%)	−8% (−15 to −1)
**AFB smear-positive at baseline**				
Success versus all other outcomes	205/297	142/248	195 (72%)	15% (5 to 24)
Survival versus death	224/297	186/248	195 (72%)	3% (−6 to 12)
Disease-free survival versus survival with treatment failure or recurrence	223/224	177/186	135 (66%)	5% (1 to 9)
Loss to follow-up versus all other outcomes	18/297	35/248	195 (72%)	−9% (−15 to −3)
**AFB smear-negative at baseline**				
Success versus all other outcomes	224/307	189/322	209 (66%)	17% (8 to 26)
Survival versus death	244/307	230/322	209 (66%)	13% (5 to 22)
Disease-free survival versus survival with treatment failure or recurrence	242/244	223/230	143 (60%)	−1% (−3 to 2)
Loss to follow-up versus all other outcomes	18/307	34/322	209 (66%)	−4% (−9 to 0)
**Previously treated with first-line drugs**				
Success versus all other outcomes	181/274	153/286	186 (66%)	14% (4 to 24)
Survival versus death	198/274	188/286	186 (66%)	10% (0 to 19)
Disease-free survival versus survival with treatment failure or recurrence	196/198	181/188	124 (64%)	2% (−2 to 7)
Lost to Follow-up vs. All Other Outcomes	15/274	28/286	186 (66%)	−3% (−8 to 3)
**Never previously treated for tuberculosis**				
Success versus all other outcomes	297/414	243/413	295 (71%)	12% (4 to 19)
Survival versus death	328/414	312/413	295 (71%)	6% (−1 to 13)
Disease-free survival versus survival with treatment failure or recurrence	326/328	302/312	211 (66%)	2% (−0 to 5)
Loss to follow-up versus all other outcomes	29/414	59/413	295 (71%)	−4% (−9 to 0)

All estimates were derived from a model in which patients were matched exactly on HIV, ART, previous treatment, and baseline smear and culture results, and propensity score matched on age, sex, province of treatment, and isoniazid resistance. AFB=acid fast bacilli. PLHIV=people living with HIV.

**Figure 2:**
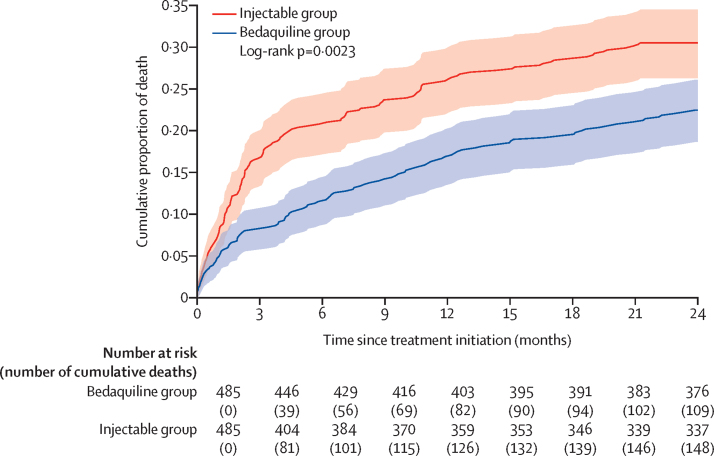
Cumulative proportion of death up to 24 months after treatment initiation in the matched population (n=485 in each treatment group) Shaded regions represent 95% CI.

A total of 361 deaths occurred within 24 months of treatment initiation. Among the 162 (24%) of 688 patients in the bedaquiline group who died within 24 months of treatment initiation, median time to death was 4·9 months (IQR 1·2–11·0); and among the 199 (28%) of 699 patients who died in the injectable group, the median time to death was 2·5 months (1·0–8·8; p=0·026 by Kruskal Wallis). Among the matched population (485 patients in each group), the cumulative proportion of death was significantly higher in the injectable group (figure 2). In the total population, findings were similar. Risk of mortality up to 24 months post treatment initiation was significantly lower in the bedaquiline group compared with the injectable group (aRD –8%; 95% CI –11 to –5; table 3). In the bedaquiline group, 117 (17%) of 688 patients died during treatment compared with 159 (23%) of 699 patients in the injectable group (–8%; –11 to –4). Post-treatment, 45 (7%) patients in the bedaquiline group died compared with 40 (6%) in the injectable group (0%; –1 to 2).

**Table 3:** Risk factors for death during treatment versus post-treatment from multivariable analysis of the entire population (n=688 bedaquiline group; n=699 injectable group)

		**Survived (n=1026)**	**Died during treatment (n=276)**	**Died after treatment (n=85)**	**Adjusted risk difference, % (95% CI)**		
					Death at any timepoint versus survival	Death during treatment versus survival	Death post-treatment versus survival
Regimen							
	Injectable group (n=699)	500 (72%)	159 (23%)	40 (6%)	0 (ref)	0 (ref)	0 (ref)
	Bedaquiline group (n=688)	526 (76%)	117 (17%)	45 (7%)	−8% (−11 to −5)	−8% (−11 to −4)	0% (−1 to 2)
Age*		36 (29 to 46)	40 (33 to 51)	35 (29 to 45)	5% (3 to 6)	4% (3 to 5)	1% (0 to 2)
Sex							
	Female (n=575)	431 (75%)	108 (19%)	36 (6%)	0 (ref)	0 (ref)	0 (ref)
	Male (n=812)	595 (73%)	168 (21%)	49 (6%)	0 (−3 to 4)	0 (−3 to 3)	−1% (−2 to 0)
AFB smear at baseline							
	Negative (n=629)	474 (75%)	118 (19%)	37 (6%)	0 (ref)	0 (ref)	0 (ref)
	Positive (n=545)	410 (75%)	104 (19%)	31 (6%)	2% (−2 to 5)	2% (−2 to 5)	−2% (−3 to 0)
	Unknown (n=213)	142 (67%)	54 (25%)	17 (8%)	6% (1 to 11)	4% (−1 to 8)	2% (−2 to 6)
Culture at baseline							
	Negative (n=254)	204 (80%)	42 (17%)	8 (3%)	0 (ref)	0 (ref)	0 (ref)
	Positive (n=676)	507 (75%)	119 (18%)	50 (7%)	7% (3 to 10)	2% (−1 to 6)	4% (3 to 6)
	Unknown (n=457)	315 (69%)	115 (25%)	27 (6%)	9% (3 to 15)	4% (−1 to 9)	2% (0 to 4)
HIV status							
	Negative (n=420)	326 (78%)	74 (18%)	20 (5%)	0 (ref)	0 (ref)	0 (ref)
	Positive on antiretroviral therapy (n=918)	674 (73%)	182 (20%)	62 (7%)	6% (3 to 10)	2% (−1 to 5)	1% (0 to 2)
	Positive not on antiretroviral therapy (n=49)	26 (53%)	20 (41%)	3 (6%)	22% (6 to 38)	18% (2 to 34)	5% (−4 to 15)
Previous treatment							
	Never treated (n=827)	640 (77%)	146 (18%)	41 (5%)	0 (ref)	0 (ref)	0 (ref)
	Treated with first-line drugs (n=560)	386 (69%)	130 (23%)	44 (8%)	8% (4 to 12)	5% (2 to 8)	2% (0 to 5)
Isoniazid resistance							
	Susceptible (n=518)	377 (73%)	108 (21%)	33 (6%)	0 (ref)	0 (ref)	0 (ref)
	Resistant (n=553)	430 (78%)	86 (16%)	37 (7%)	−4% (−8 to 1)	−3% (−6 to 1)	0% (−2 to 1)
	Unknown (n=316)	219 (69%)	82 (26%)	15 (5%)	2% (−4 to 9)	3% (−3 to 8)	−1% (−2 to 1)

Data are n (%), unless indicated otherwise. Comparisons were done only on patients who were alive during the analytical period (eg, analyses of post-treatment death versus survival were only done on patients who were alive at their treatment end date). Percentages are calculated by row, not by column; percentages might not sum up to 100% due to rounding. Model is adjusted for all factors listed in this table, including the province in South Africa where the patient initiated treatment. AFB=acid fast bacilli.

* In this row, data are median (IQR) or risk difference (95% CI) per 10-year increase in age.

Sex and isoniazid resistance were not associated with risk of mortality. HIV and ART status, previous tuberculosis treatment, and increasing age were significantly associated with mortality during treatment. The characteristics associated with post-treatment mortality were baseline culture, increasing age, and history of previous tuberculosis treatment (table 3).

When stratifying the analysis according to treatment received (appendix 2 pp 8–9), we found similar findings, except that in people receiving a bedaquiline-containing regimen, risk of death was not greater among PLHIV receiving ART than people who were HIV negative.

In sensitivity analysis evaluating death within the first three months of treatment versus after three months, 171 (47%) of 361 deaths (63 in the bedaquiline group and 108 in the injectable group) happened within the first three months of treatment initiation, and 190 (53%) deaths (99 in the bedaquiline group and 91 in the injectable group) happened after three months of treatment. Findings from regression analyses were qualitatively similar to those of on-treatment mortality versus post-treatment mortality (figure 2, appendix 2 pp 10–12).

## Discussion

We measured and compared treatment outcomes for patients with rifampicin-resistant tuberculosis treated with a short, bedaquiline-containing regimen and patients treated with a short, injectable-containing regimen. We found that the bedaquiline-containing regimen was associated with higher probability of treatment success 24 months after treatment initiation, lower risk of loss to follow-up, and lower risk of mortality during treatment. Importantly, PLHIV receiving ART did not appear to be at increased risk of mortality compared with HIV negative patients in the bedaquiline group.

Poor outcomes in the context of rifampicin-resistant tuberculosis treatment are driven by high death rates and high losses to follow-up.^16^, ^17^ Indeed, loss to follow-up is one of the two major challenges experienced by the South Africa National TB Programme.^10^ A study by Abidi and colleagues^18^ found that the major benefit of shorter rifampicin-resistant tuberculosis treatment regimens is the lower loss to follow-up rate. Therefore, it is interesting that the bedaquiline group had significantly lower risk of loss to follow-up compared with the injectable group receiving treatment of the same duration. One explanation for this finding is that bedaquiline is better tolerated than injectables, leading to better treatment adherence.^19^

The reduction in mortality associated with bedaquiline has previously been demonstrated in longer rifampicin-resistant tuberculosis treatment regimens.^16^ Although we recognise that mortality remains high in our cohort, which we speculate might be due to advanced HIV or delayed health-care seeking, diagnosis, or both, which results in advanced disease at diagnosis.^20^ Another study conducted in South Africa showed increased treatment success rate with bedaquiline among patients with rifampicin-resistant tuberculosis on longer treatment regimens.^21^ It appears that the same benefit might be achieved using shorter treatment regimens, particularly in the first three months of treatment, as highlighted in this analysis. This finding is consistent with a meta-analysis^22^ that established that the use of injectable agents was associated with higher mortality among patients with rifampicin-resistant tuberculosis and other meta-analyses^22^, ^23^ that suggested bedaquiline has the potential to achieve a higher sputum culture conversion rate and a lower mortality risk among patients with rifampicin-resistant tuberculosis compared with patients not receiving bedaquiline. A study by Tack and colleagues^24^ suggests an all-oral, bedaquiline-based regimen that includes linezolid is safe and effective; however, the study was not comparative and it remains to be elucidated if such a regimen would be superior to the bedaquiline-containing regimen (without linezolid) in this analysis.

The findings of increased risk of unfavourable rifampicin-resistant tuberculosis treatment outcomes among PLHIV who were not on ART further supports the need to establish HIV infection status and initiate ART. Furthermore, our findings of similar risk of mortality among HIV-negative people and PLHIV on ART in the bedaquiline group support the use of bedaquiline among PLHIV on ART and reinforce the importance of timely initiation of ART regimens among PLHIV.^25^

The bedaquiline-containing regimen presented in this study has a significant pill burden; however, its shorter length is likely to make it preferable than a regimen with a smaller daily pill burden lasting 18–20 months.^3^ Further, it is also likely to be preferred over the injectable-containing short regimen in this study, as it replaces the injectable agent—which is difficult to administer and associated with ototoxicity and other side-effects—with an oral medication.

The South African National HIV/TB strategic plan provides a target of 75% treatment success rate for all rifampicin-resistant tuberculosis or multidrug-resistant tuberculosis by 2022.^26^ Despite substantially improved outcomes in the bedaquiline group in this study, additional research is required to further improve regimen effectiveness, safety, and completion for all patients with rifampicin-resistant tuberculosis. This research might include substitution of more effective drugs into the short regimen, or the use of novel drugs and regimens, such as those containing delamanid or pretomanid.^27^

Our study has several limitations. It is based on observational data that cannot determine physician's reasoning to start someone on a particular regimen and might not capture all important patient characteristics, which could cause residual confounding. Different provinces had differential roll out of bedaquiline. We attempted to account for differences between patients treated across provinces by including it as a variable in propensity score matching. The assessment of outcomes post-treatment required a valid national ID to be registered, which might lead to selection bias. Although this analysis included many patients, they were all from South Africa and a majority had HIV-coinfection. Further research is required to ensure our findings are generalisable, although stratified analyses did not identify important differences in outcomes among PLHIV and people without HIV. We did not assess safety of the regimens in this study, however previous analyses have shown bedaquiline is safer^19^, ^24^, ^28^, ^29^, ^30^ and associated with substantially lower rates of treatment discontinuation than injectables.^19^ Finally, due to the nature of programmatic data, there might be misclassification bias of patients in the registry who were initially started on a short regimen and had to switch to a long regimen. This misclassification would lead to under-ascertainment of treatment failure, however all available evidence previous to this study suggests regimen switches would be more common with injectable-containing regimens.^10^

A major strength of our study is the 24-month follow-up of patients initiating short rifampicin-resistant tuberculosis regimens in routine conditions. The results reported in this study led to new WHO recommendations for the use of bedaquiline in shorter rifampicin-resistant tuberculosis treatment regimens.^31^ The use of national identification numbers enabled an analysis of recurrence and mortality in the year following treatment completion. This analysis indicated that both regimens were associated with low rates of recurrence (0·2% bedaquiline *vs* 1% injectables), similar to those observed in a multinational study of the injectable-containing regimen,^32^ while also finding that post-treatment mortality was common and similar in the two treatment groups (7·9% bedaquiline *vs* 7·4% injectables). The availability of individual-level data allowed adjustment for potential confounders and helped reduce biases that could emerge from identifiable patient and clinical characteristics. These findings add to the literature regarding effectiveness of shorter rifampicin-resistant tuberculosis treatment regimens**.**

The use of bedaquiline instead of an injectable in a shorter rifampicin-resistant tuberculosis treatment regimen is associated with significant improvement in treatment success 24 months after treatment initiation, and significantly lower mortality and loss to follow-up during treatment. Bedaquiline could replace injectables in short regimens for all patients who are eligible, while efforts continue to further improve the effectiveness, safety, and completion of rifampicin-resistant tuberculosis regimens.

## Data sharing

The data used for this analysis in the form of deidentified participant data and a data dictionary will be made available after publication. Investigators wishing to access these data will need to have an approved research proposal and complete a data access agreement. All inquiries should be sent to the corresponding author (Norbert.Ndjeka@health.gov.za or norbertndjeka@gmail.com).

## Declaration of interests

We declare no competing interests.

## Supplementary Materials

French translation of the abstract
