# Supplementary material for: Treatment outcomes 24 months after initiating short, all-oral bedaquiline-containing or injectable-containing rifampicin-resistant tuberculosis treatment regimens in South Africa: a retrospective cohort study
Source: Lancet Infect Dis. 2022 Jul;22(7):1042–51. doi: 10.1016/S1473-3099(21)00811-2 (PMC9217754; doi:10.1016/S1473-3099(21)00811-2)
Supplement: French translation of the abstract [file mmc1.pdf]

THE LANCET
Infectious Diseases

Supplementary appendix 1

This translation in French was submitted by the authors and we reproduce it as supplied. It has not been peer reviewed. *The Lancet*'s editorial processes have only been applied to the original in English, which should serve as reference for this manuscript.

Supplement to: Ndjeka N, Campbell JR, Meintjes G, et al. Treatment outcomes 24 months after initiating short bedaquiline-containing or injectable-containing rifampicin-resistant tuberculosis treatment regimens in South Africa: a retrospective cohort study. *Lancet Infect Dis* 2022; published online May 2. https://doi.org/10.1016/S1473-3099(21)00811-2.

Cette traduction en français a été proposée par les auteurs et nous l'avons reproduite telle quelle. Elle n'a pas été examinée par des pairs. Les processus éditoriaux du *Lancet* n'ont été appliqués qu'à l'original en anglais et c'est cette version qui doit servir de référence pour ce manuscrit.

Résumé

Contexte Il existe un besoin pour un traitement oral court et sûr de la tuberculose résistante à la rifampicine. Nous avons comparé jusqu'à 24 mois après le début du traitement chez les patients atteints de tuberculose résistante à la rifampicine en Afrique du Sud. Ils ont été traités avec un régime court contenant de la bédaquiline par voie orale (groupe bédaquiline) ou un régime court contenant un agent injectable dans le régime (groupe injectable).

Méthodes Les patients atteints de tuberculose résistante à la rifampicine, âgés de 18 ans ou plus, éligibles à un régime de courte durée commençant le traitement entre le 1er janvier et le 31 décembre 2017, avec un régime contenant de la bédaquiline ou un injectable recommandé par l'OMS dans un schéma thérapeutique de 9 à 12 mois, enregistré dans la base de données sur la tuberculose résistante aux médicaments (EDRWeb), et dont l'âge, le sexe, le statut VIH et le numéro d'identification national connus étaient admissibles aux critères d'inclusions dans l'étude; les patients ayant reçu du linézolide, des carbapénèmes, de la terizidone ou de la cyclosérine, du délamanide ou de l'acide para-amino-salicylique ont été exclus. La bédaquiline a été administrée à une dose de 400 mg une fois par jour pendant deux semaines, suivie de 200 mg trois fois par semaine pendant 22 semaines. Pour comparer les schémas thérapeutiques, les patients ont été exactement appariés sur le statut VIH et ART, la tuberculose antérieure, les antécédents de traitement et le résultat de frottis et de culture de bacilles acido-résistants de base, tandis que le score de propension apparié à l'âge, au sexe, la province de traitement, et le statut de sensibilité à l'isoniazide. Nous avons effectué une régression linéaire binomiale pour estimer les différences de risque ajusté (aRD) et IC à 95 % pour les critères de jugement à 24 mois, qui comprenaient : le succès du traitement (c.-à-d. guérison ou achèvement du traitement sans preuve de récidive) par rapport à tous les autres critères de jugement, la survie par rapport au décès, la survie sans maladie par rapport à la survie en cas d'échec ou de récidive du traitement et à la perte de suivi par rapport à tous les autres résultats.

Résultats Dans l'ensemble, 1387 (14 %) des 10152 patients atteints de tuberculose résistante à la rifampicine traités en 2017 ont rencontré les critères d'inclusion; 688 dans le groupe bédaquiline et 699 dans le groupe injectable. Quatre patients (1 %) ont eu un échec de traitement ou une récidive, 44 (6 %) ont été perdus de vue et 162 (24 %) sont décédés dans le groupe bédaquiline, comparativement à 17 (2 %), 87 (12 %) et 199 (29 %), respectivement, dans le groupe injectable. Dans les analyses ajustées, le succès du traitement était de 14 % (IC à 95 % de 8 à 20) plus élevé dans le groupe bédaquiline que dans le groupe injectable (70 % *vs* 57 %); les pertes de vue étaient de 4 % (1 à 8) plus faible dans le groupe bédaquiline (6 % *vs* 12 %); et la survie sans maladie était de 2 % (0 à 5) plus élevée chez le groupe bédaquiline (99 % vs 97 %). Le groupe bédaquiline avait un risque de mortalité inférieur de 8% (4 à 11) pendant le traitement (17,0 % *vs* 22,4 %), mais il n'y avait pas de différence dans la mortalité après le traitement.

Interprétation Les patients du groupe bédaquiline ont connu des taux significativement plus élevés de succès du traitement à 24 mois. Cette découverte soutient l'utilisation de régimes courts contenant de la bédaquiline chez les patients éligibles.

Financement Programme mondial de lutte contre la tuberculose de l'OMS.
