# Supplementary material for: Treatment outcomes 24 months after initiating short, all-oral bedaquiline-containing or injectable-containing rifampicin-resistant tuberculosis treatment regimens in South Africa: a retrospective cohort study
Source: Lancet Infect Dis. 2022 Jul;22(7):1042–51. doi: 10.1016/S1473-3099(21)00811-2 (PMC9217754; doi:10.1016/S1473-3099(21)00811-2)
Supplement: Supplementary appendix 2 [file mmc2.pdf]

THE LANCET
Infectious Diseases

Supplementary appendix 2

This appendix formed part of the original submission and has been peer reviewed. We post it as supplied by the authors.

Supplement to: Ndjeka N, Campbell JR, Meintjes G, et al. Treatment outcomes 24 months after initiating short bedaquiline-containing or injectable-containing rifampicin-resistant tuberculosis treatment regimens in South Africa: a retrospective cohort study. *Lancet Infect Dis* 2022; published online May 2. https://doi.org/10.1016/S1473-3099(21)00811-2.

Appendix to: Treatment outcomes 24 months after initiating short, all-oral bedaquiline-containing or injectable-containing rifampicin-resistant tuberculosis treatment regimens in South Africa: a retrospective cohort study

Norbert Ndjeka, Jonathon R Campbell, Graeme Meintjes, Gary Maartens, H Simon Schaaf, Jennifer Hughes, Xavier Padanilam, Anja Reuter, Rodolfo Romero, Farzana Ismail, Martin Enwerem, Hannetjie Ferreira, Francesca Conradie, Kogieleum Naidoo, Dick Menzies

Contents

Appendix Table 1: Distribution of included patients and select characteristics by province of treatment 2

Appendix Table 2: Characteristics of patients eligible for and receiving a short regimen who were ultimately included vs excluded (N=2769) 3

Appendix Table 3: Univariable analysis results to support decisions on exact and propensity score matching in multivariable analysis 4

Appendix Figure 1: Love Plot of Changes in Standardized Mean Difference Before and After Matching with Caliper of 0.5 5

Appendix Figure 2: Love Plot of Changes in Standardized Mean Difference Before and After Matching with Caliper of 0.2 6

Appendix Table 4: Primary Analysis Outcomes from Multivariable Analysis using caliper distance of 0.2 7

Appendix Table 5: Risk of mortality during or post-treatment estimated via multivariable analysis among patients in the injectable group 8

Appendix Table 6: Risk of mortality during or post-treatment estimated via multivariable analysis among patients in the bedaquiline group 9

Appendix Table 7: Factors for mortality by early vs. late death as estimated by multivariable analysis 10

Appendix Table 8: Risk of early or late mortality as estimated by multivariable analysis among patients in the injectable group 11

Appendix Table 9: Risk of early or late mortality as estimated by multivariable analysis among patients in the bedaquiline group 12

Appendix Table 1: Distribution of included patients and select characteristics by province of treatment

Province	Regimen Group	N	Success, %	Loss to Follow-up, %	PLHIV, %	PLHIV Receiving ART, %
Eastern Cape	Bedaquiline	201	75	5	64	98
	Injectable	230	54	13	66	97
Free State	Bedaquiline	31	71	3	74	96
	Injectable	20	50	5	65	85
Gauteng	Bedaquiline	63	62	14	79	98
	Injectable	24	54	21	67	94
Kwazulu-Natal	Bedaquiline	163	75	6	74	100
	Injectable	232	59	14	72	95
Limpopo	Bedaquiline	16	62	6	62	100
	Injectable	11	55	0	64	71
Mpumalanga	Bedaquiline	99	66	8	85	92
	Injectable	70	57	10	77	93
North West	Bedaquiline	75	61	9	72	94
	Injectable	41	63	15	66	63
Northern Cape	Bedaquiline	29	59	0	55	100
	Injectable	30	37	20	67	95
Western Cape	Bedaquiline	11	55	0	64	100
	Injectable	41	66	17	44	94

Appendix Table 2: Characteristics of patients eligible for and receiving a short regimen who were ultimately included vs excluded (N=2769)

	Included	Excluded	p-value
N	1387	1382	--
**Patient Characteristics**			
Median (IQR) Age	37 (30 to 47)	36 (30 to 44)	0.0417
Male	812 (58.5%)	798 (57.7%)	0.6974
HIV Infection	967 (69.7%)	1013 (73.3%)	0.0409
If HIV Infected, on ART	918 (94.9%)	950 (93.8%)	0.3116
**Microbiologic Findings at Baseline***			
If Result Available, AFB Smear Positive at Baseline	545 (46.4%)	563 (49.6%)	0.1423
If Result Available, Culture Positive at Baseline	676 (72.7%)	722 (77.9%)	0.0110
If Result Available, Culture or AFB or Xpert Positive at Baseline	1207 (91.2%)	1174 (90.8%)	0.7956
**Previous Treatment**			
Previously Treated for TB with First Line Drugs	560 (40.4%)	577 (41.8%)	0.4856
**Treatment Received**			
Received Moxifloxacin or Levofloxacin	1387 (100%)	1349 (97.6%)	--
Received Bedaquiline	688 (49.6%)	946 (68.5%)	--
Received Linezolid	0 (0%)	160 (11.6%)	--
Received Cycloserine/Terizidone	0 (0%)	211 (15.3%)	--
Received Clofazimine	1387 (100%)	1271 (92%)	--
Received Injectable	699 (50.4%)	968 (70%)	--
Received Amikacin	106 (7.6%)	115 (8.3%)	--
Received Kanamycin	595 (42.9%)	854 (61.8%)	--
Received Capreomycin	1 (0.1%)	3 (0.2%)	--
Received Streptomycin	0 (0%)	0 (0%)	--
Mean Number of Other Drugs Received	2.9	2.9	--
**End of Treatment Outcomes**			
Number with Treatment Success	928	922	0.9466
Number with Failure	19	25	0.4402
Number Dying During Treatment	276	250	0.2440
Number Lost to follow-up During Treatment	164	185	0.2375

Abbreviations: AFB, acid fast bacilli; ART, antiretroviral treatment; IQR, interquartile range; TB, tuberculosis

Notes: p-value calculated using Kruskal-Wallis test for medians and chi-square test for continuous variables

Appendix Table 3: Univariable analysis results to support decisions on exact and propensity score matching in multivariable analysis

Characteristic	Success/Total	Risk Difference per 100 (95% CI)
Age, median (IQR), per 10y increase	37 (30 to 47)	-1 (-3 to 1)
Sex		
Female	373/575	0 (reference)
Male	501/812	-3 (-8 to 2)
AFB Smear at Baseline		
Negative	413/629	0 (reference)
Positive	347/545	-2 (-7 to 3)
Unknown	114/213	-12 (-20 to -4)
Culture at Baseline		
Negative	182/254	0 (reference)
Positive	427/676	-8 (-15 to -2)
Unknown	265/457	-14 (-21 to -7)
HIV-Status		
Negative	275/420	0 (reference)
Positive on ART	582/918	-2 (-8 to 3)
Positive not on ART	17/49	-31 (-45 to -17)
Previous Treatment		
Never Treated	540/827	0 (reference)
Treated with First Line Drugs	334/560	-6 (-11 to -1)
Isoniazid Resistance		
Susceptible	325/518	0 (reference)
Resistant	362/553	3 (-3 to 8)
Unknown	187/316	-4 (-10 to 3)

Abbreviations: IQR, interquartile range; AFB, acid fast bacilli; ART, antiretroviral therapy; 95% CI, 95% confidence interval.

Appendix Figure 1: Love Plot of Changes in Standardized Mean Difference Before and After Matching with Caliper of 0.5

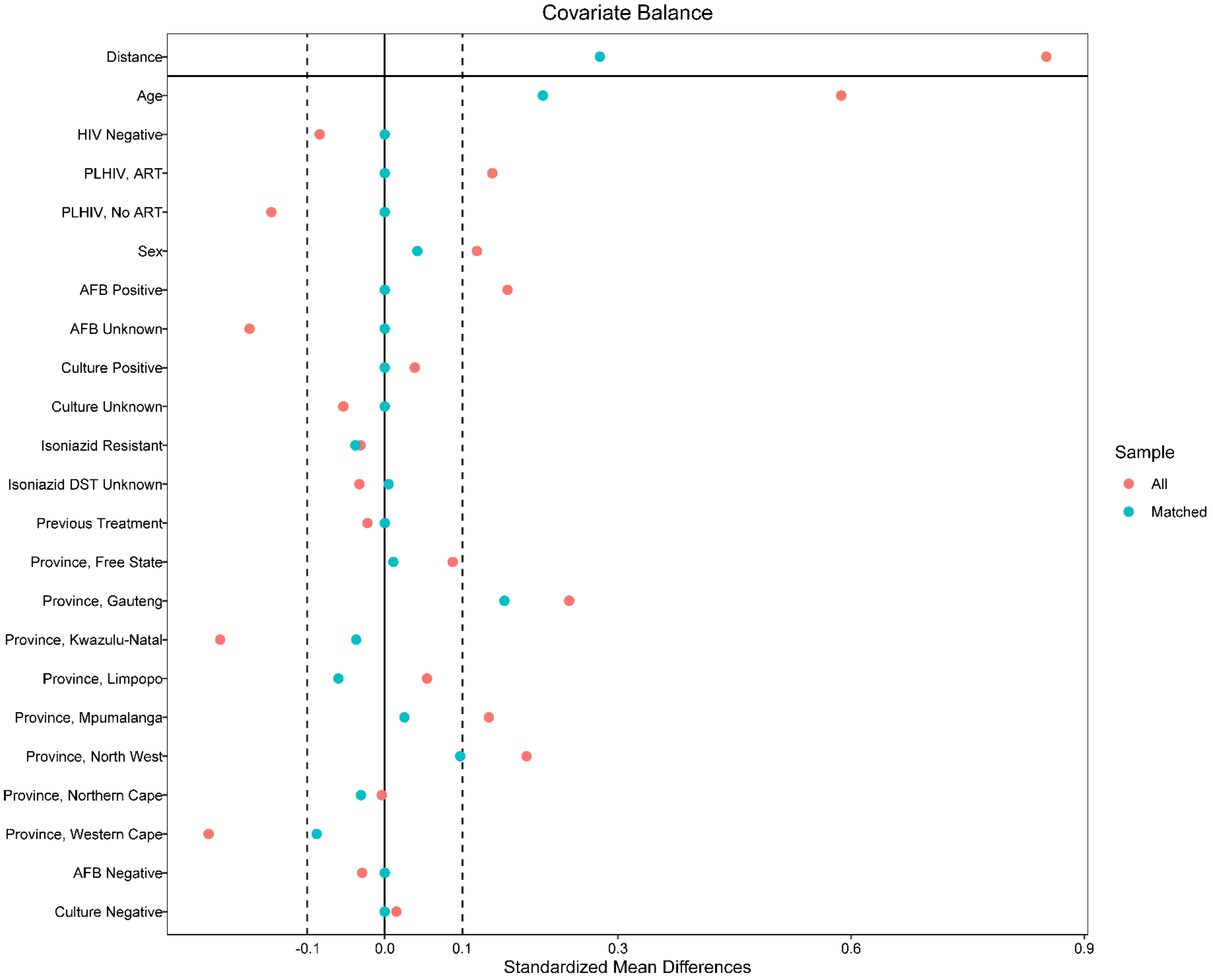

Appendix Figure 2: Love Plot of Changes in Standardized Mean Difference Before and After Matching with Caliper of 0.2

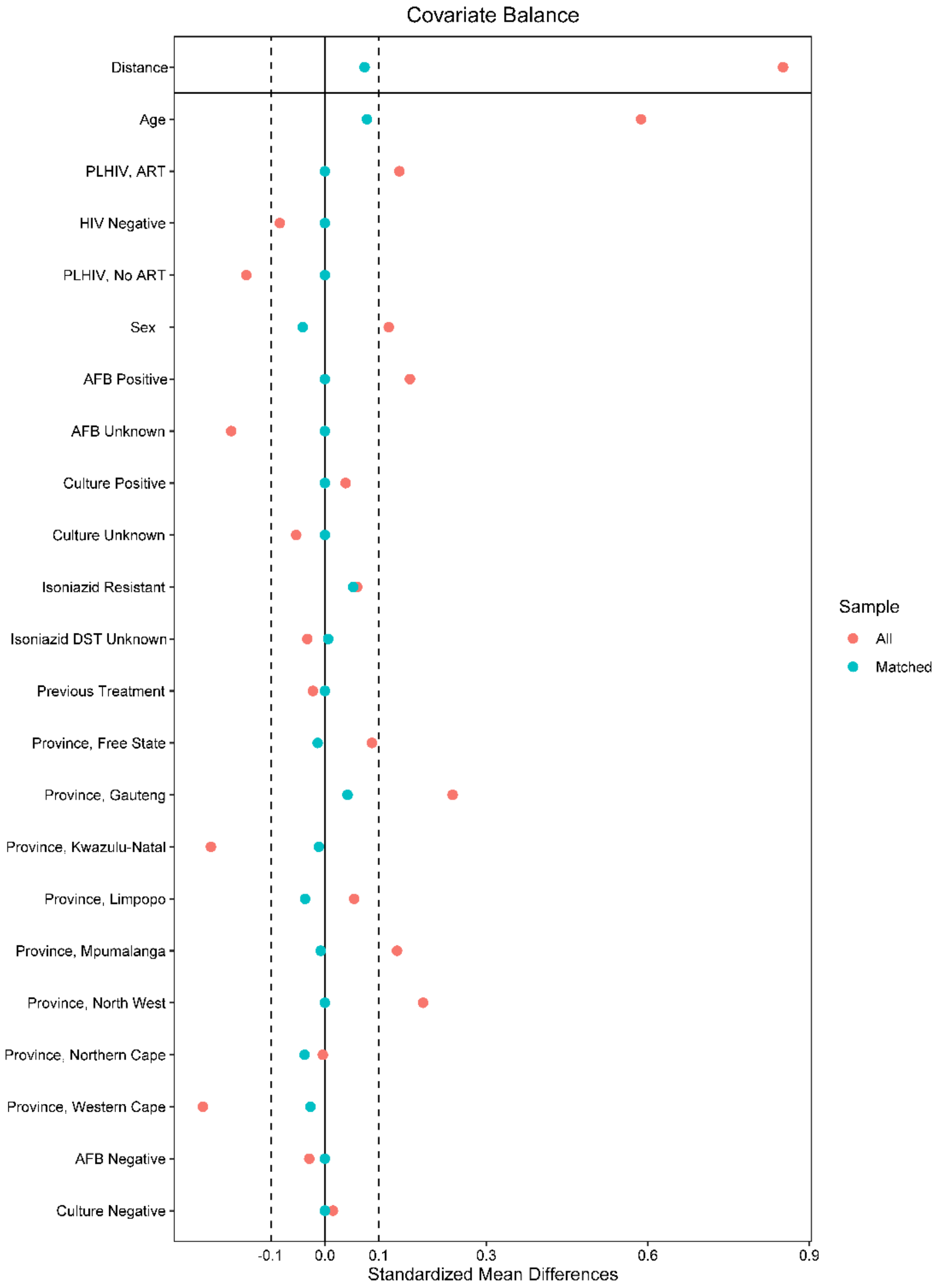

Appendix Table 4: Primary Analysis Outcomes from Multivariable Analysis using caliper distance of 0.2

Outcome	Bedaquiline Group (events/total)	Injectable Group (events/total)	Matched Pairs (% Matched)	Adjusted RD per 100 (95% CI)
Total Population				
Success vs. All Other Outcomes	478/688	396/699	394 (57%)	16 (10, 23)
Survival vs. Death	526/688	500/699	394 (57%)	10 (4, 16)
Disease-Free Survival vs. Survival with Treatment Failure/Recurrence	522/526	483/500	281 (55%)	2 (-0.1, 4)
Lost to Follow-up vs. All Other Outcomes	478/688	396/699	394 (57%)	-6 (-10, -2)
PLHIV Receiving ART				
Success vs. All Other Outcomes	337/478	245/440	289 (63%)	18 (10, 26)
Survival vs. Death	372/478	302/440	289 (63%)	13 (6, 21)
Disease-Free Survival vs. Survival with Treatment Failure/Recurrence	368/372	296/302	197 (58%)	1 (-1, 3)
Lost to Follow-up vs. All Other Outcomes	31/478	51/440	289 (63%)	-4 (-9, 1)
People HIV-Negative				
Success vs. All Other Outcomes	138/195	137/225	105 (50%)	19 (6, 32)
Survival vs. Death	147/195	179/225	105 (50%)	6 (-6, 17)
Disease-Free Survival vs. Survival with Treatment Failure/Recurrence	147/147	169/179	71 (44%)	6 (0.3, 11)
Lost to Follow-up vs. All Other Outcomes	9/195	32/225	105 (50%)	-9 (-16, -1)
AFB Smear Positive at Baseline				
Success vs. All Other Outcomes	205/297	142/248	170 (62%)	15 (5, 25)
Survival vs. Death	224/297	186/248	170 (62%)	6 (-3, 16)
Disease-Free Survival vs. Survival with Treatment Failure/Recurrence	223/224	177/186	108 (53%)	5 (1, 9)
Lost to Follow-up vs. All Other Outcomes	18/297	35/248	170 (62%)	-5 (-11, 1)
AFB Smear Negative at Baseline				
Success vs. All Other Outcomes	224/307	189/322	173 (55%)	16 (6, 25)
Survival vs. Death	244/307	230/322	173 (55%)	16 (7, 25)
Disease-Free Survival vs. Survival with Treatment Failure/Recurrence	242/244	223/230	118 (50%)	0 (-2, 2)
Lost to Follow-up vs. All Other Outcomes	18/307	34/322	173 (55%)	0 (-6, 6)
Previously Treated with First-Line Drugs				
Success vs. All Other Outcomes	181/274	153/286	158 (56%)	11 (1, 22)
Survival vs. Death	198/274	188/286	158 (56%)	9 (-1, 19)
Disease-Free Survival vs. Survival with Treatment Failure/Recurrence	196/198	181/188	97 (50%)	4 (-1, 9)
Lost to Follow-up vs. All Other Outcomes	15/274	28/286	158 (56%)	-1 (-7, 4)
Never Previously Treated for Tuberculosis				
Success vs. All Other Outcomes	297/414	243/413	245 (59%)	14 (6, 22)
Survival vs. Death	328/414	312/413	245 (59%)	7 (-1, 14)
Disease-Free Survival vs. Survival with Treatment Failure/Recurrence	326/328	302/312	178 (56%)	2 (-1, 5)
Lost to Follow-up vs. All Other Outcomes	29/414	59/413	245 (59%)	-7 (-12, -2)

Abbreviations: RD, risk difference; AFB, acid fast bacilli; ART, antiretroviral therapy; CI, confidence interval. Note: Estimates comes from a model where patients were matched exactly on HIV, ART, previous treatment, and baseline smear and culture results, while propensity score matched on age, sex, province of treatment, and isoniazid resistance.

Appendix Table 5: Risk of mortality during or post-treatment estimated via multivariable analysis among patients in the injectable group

Characteristic	Survived (n=500)	Death During Treatment (n=159)	Death Post-Treatment (n=40)	aRD per 100 (95%CI) Death Ever vs. Survival	aRD per 100 (95%CI) Death During Treatment vs. Survival	aRD per 100 (95%CI) Death Post-Treatment vs. Survival
Age, median (IQR), per 10y	33 (27 to 40)	38 (31 to 47)	33 (27 to 40)	6 (3 to 9)	6 (4 to 8)	0.4 (0.01 to 1)
Sex						
Female	228 (73.5%)	63 (20.3%)	19 (6.1%)	0 (reference)	0 (reference)	0 (reference)
Male	272 (69.9%)	96 (24.7%)	21 (5.4%)	1 (-4 to 7)	2 (-1 to 6)	-1 (-2 to 1)
AFB Smear at Baseline						
Negative	230 (71.4%)	72 (22.4%)	20 (6.2%)	0 (reference)	0 (reference)	0 (reference)
Positive	186 (75%)	52 (21%)	10 (4%)	-1 (-7 to 5)	2 (-2 to 5)	-2 (-4 to 0.4)
Unknown	84 (65.1%)	35 (27.1%)	10 (7.8%)	3 (-8 to 14)	1 (-7 to 10)	1 (-3 to 6)
Culture at Baseline						
Negative	96 (76.2%)	26 (20.6%)	4 (3.2%)	0 (reference)	0 (reference)	0 (reference)
Positive	244 (73.1%)	69 (20.7%)	21 (6.3%)	8 (1 to 14)	4 (-0.2 to 9)	2 (-1 to 4)
Unknown	160 (66.9%)	64 (26.8%)	15 (6.3%)	9 (1 to 18)	6 (-1 to 13)	3 (0.2 to 5)
HIV-Status						
Negative	179 (79.6%)	39 (17.3%)	7 (3.1%)	0 (reference)	0 (reference)	0 (reference)
Positive on ART	302 (68.6%)	107 (24.3%)	31 (7%)	8 (2 to 15)	6 (2 to 10)	2 (0.1 to 4)
Positive not on ART	19 (55.9%)	13 (38.2%)	2 (5.9%)	22 (4 to 41)	18 (1 to 35)	0 (-3 to 2)
Previous Treatment						
Never Treated	312 (75.5%)	84 (20.3%)	17 (4.1%)	0 (reference)	0 (reference)	0 (reference)
Treated with First Line Drugs	188 (65.7%)	75 (26.2%)	23 (8%)	8 (1 to 15)	4 (-1 to 9)	2 (-0.3 to 5)
Isoniazid Resistance						
Susceptible	177 (70.5%)	56 (22.3%)	18 (7.2%)	0 (reference)	0 (reference)	0 (reference)
Resistant	217 (76.4%)	51 (18%)	16 (5.6%)	-2 (-8 to 5)	-3 (-8 to 2)	0 (-3 to 3)
Unknown	106 (64.6%)	52 (31.7%)	6 (3.7%)	7 (-1 to 15)	8 (1 to 15)	-2 (-4 to 1)

Abbreviations: AFB, acid fast bacilli; aRD, adjusted risk difference; 95% CI, 95% confidence interval; ART, antiretroviral treatment; IQR, interquartile range

*Notes: Comparisons were done only on patients who were alive during the analytical period (e.g., analyses of post-treatment death vs. survival were only done on patients who were alive at their treatment end date). Percentages represent row percentages. Model is adjusted for all factors listed here, including province of treatment.

Appendix Table 6: Risk of mortality during or post-treatment estimated via multivariable analysis among patients in the bedaquiline group

Characteristic	Survived (n=526)	Death During Treatment (n=117)	Death Post Treatment (n=45)	aRD per 100 (95%CI) Death Ever vs. Survival	aRD per 100 (95%CI) Death During Treatment vs. Survival	aRD per 100 (95%CI) Death Post-Treatment vs. Survival
Age, median (IQR), per 10y	41 (33 to 51)	44 (35 to 57)	38 (31 to 56)	4 (2 to 6)	4 (2 to 5)	0 (-1 to 1)
Sex						
Female	203 (76.6%)	45 (17%)	17 (6.4%)	0 (reference)	0 (reference)	0 (reference)
Male	323 (76.4%)	72 (17%)	28 (6.6%)	0 (-5 to 4)	-2 (-5 to 1)	-1 (-3 to 0.1)
AFB Smear at Baseline						
Negative	244 (79.5%)	46 (15%)	17 (5.5%)	0 (reference)	0 (reference)	0 (reference)
Positive	224 (75.4%)	52 (17.5%)	21 (7.1%)	3 (-3 to 8)	2 (-1 to 6)	-1 (-2 to 1)
Unknown	58 (69%)	19 (22.6%)	7 (8.3%)	7 (-6 to 19)	3 (-2 to 9)	2 (-3 to 7)
Culture at Baseline						
Negative	108 (84.4%)	16 (12.5%)	4 (3.1%)	0 (reference)	0 (reference)	0 (reference)
Positive	263 (76.9%)	50 (14.6%)	29 (8.5%)	8 (2 to 14)	0 (-3 to 2)	7 (4 to 10)
Unknown	155 (71.1%)	51 (23.4%)	12 (5.5%)	10 (1 to 18)	6 (-0.3 to 11)	2 (-0.1 to 3)
HIV-Status						
Negative	147 (75.4%)	35 (17.9%)	13 (6.7%)	0 (reference)	0 (reference)	0 (reference)
Positive on ART	372 (77.8%)	75 (15.7%)	31 (6.5%)	1 (-4 to 6)	-1 (-4 to 1)	-3 (-5 to -0.4)
Positive not on ART	7 (46.7%)	7 (46.7%)	1 (6.7%)	25 (-7 to 57)	16 (-16 to 48)	6 (-21 to 32)
Previous Treatment						
Never Treated	328 (79.2%)	62 (15%)	24 (5.8%)	0 (reference)	0 (reference)	0 (reference)
Treated with First Line Drugs	198 (72.3%)	55 (20.1%)	21 (7.7%)	5 (0.4 to 10)	2 (-1 to 5)	2 (-1 to 4)
Isoniazid Resistance						
Susceptible	200 (74.9%)	52 (19.5%)	15 (5.6%)	0 (reference)	0 (reference)	0 (reference)
Resistant	213 (79.2%)	35 (13%)	21 (7.8%)	-4 (-9 to 2)	-5 (-9 to -1)	1 (-1 to 2)
Unknown	113 (74.3%)	30 (19.7%)	9 (5.9%)	2 (-7 to 11)	0 (-7 to 7)	1 (-0.5 to 3)

Abbreviations: AFB, acid fast bacilli; aRD, adjusted risk difference; 95% CI, 95% confidence interval; ART, antiretroviral treatment; IQR, interquartile range

*Notes: Comparisons were done only on patients who were alive during the analytical period (e.g., analyses of post-treatment death vs. survival were only done on patients who were alive at their treatment end date). Percentages represent row percentages. Model is adjusted for all factors listed here, including province of treatment.

Appendix Table 7: Factors for mortality by early vs. late death as estimated by multivariable analysis

Characteristic	Survived (n=1026)	Early Death: died ≤3 months after treatment start (n=171)	Late Death: died >3 to 24 months after treatment start (n=190)	aRD per 100 (95%CI) Death Ever vs. Survival	aRD per 100 (95%CI) Early Death vs. Survival	aRD per 100 (95%CI) Late Death vs. Survival
Regimen						
Injectable-Containing	500 (71.5%)	108 (15.5%)	91 (13%)	0 (reference)	0 (reference)	0 (reference)
Bedaquiline-Containing	526 (76.5%)	63 (9.2%)	99 (14.4%)	-8 (-11 to -5)	-6 (-9 to -4)	0 (-3 to 3)
Age, median (IQR), per 10y	36 (29 to 46)	40 (34 to 53)	38 (31 to 49)	5 (3 to 6)	3 (2 to 3)	2 (1 to 3)
Sex						
Female	431 (75%)	68 (11.8%)	76 (13.2%)	0 (reference)	0 (reference)	0 (reference)
Male	595 (73.3%)	103 (12.7%)	114 (14%)	0 (-3 to 4)	0 (-1 to 1)	0 (-1 to 1)
AFB Smear at Baseline						
Negative	474 (75.4%)	68 (10.8%)	87 (13.8%)	0 (reference)	0 (reference)	0 (reference)
Positive	410 (75.2%)	65 (11.9%)	70 (12.8%)	2 (-2 to 5)	1 (-1 to 4)	-2 (-4 to 1)
Unknown	142 (66.7%)	38 (17.8%)	33 (15.5%)	6 (1 to 11)	3 (0.1 to 6)	4 (-2 to 9)
Culture at Baseline						
Negative	204 (80.3%)	21 (8.3%)	29 (11.4%)	0 (reference)	0 (reference)	0 (reference)
Positive	507 (75%)	71 (10.5%)	98 (14.5%)	7 (3 to 10)	-1 (-3 to 1)	4 (1 to 6)
Unknown	315 (68.9%)	79 (17.3%)	63 (13.8%)	9 (3 to 15)	3 (-0.2 to 7)	2 (-1 to 5)
HIV-Status						
Negative	326 (77.6%)	51 (12.1%)	43 (10.2%)	0 (reference)	0 (reference)	0 (reference)
Positive on ART	674 (73.4%)	104 (11.3%)	140 (15.3%)	6 (3 to 10)	0 (-2 to 1)	5 (2 to 8)
Positive not on ART	26 (53.1%)	16 (32.7%)	7 (14.3%)	22 (6 to 38)	6 (-7 to 19)	8 (-5 to 21)
Previous Treatment						
Never Treated	640 (77.4%)	99 (12%)	88 (10.6%)	0 (reference)	0 (reference)	0 (reference)
Treated with First Line Drugs	386 (68.9%)	72 (12.9%)	102 (18.2%)	8 (4 to 12)	1 (-1 to 3)	7 (3 to 11)
Isoniazid Resistance						
Susceptible	377 (72.8%)	62 (12%)	79 (15.3%)	0 (reference)	0 (reference)	0 (reference)
Resistant	430 (77.8%)	55 (9.9%)	68 (12.3%)	-4 (-8 to 1)	-1 (-3 to 0.5)	-2 (-5 to 1)
Unknown	219 (69.3%)	54 (17.1%)	43 (13.6%)	2 (-4 to 9)	0 (-3 to 4)	0 (-4 to 5)

Abbreviations: AFB, acid fast bacilli; aRD, adjusted risk difference; 95% CI, 95% confidence interval; ART, antiretroviral treatment; IQR, interquartile range

*Notes: Comparisons were done only on patients who were alive during the analytical period (e.g., analyses of death >3 months vs. survival were only done on patients who were alive after 3 months of treatment). Percentages represent row percentages. Model is adjusted for all factors listed here, including province of treatment.

Appendix Table 8: Risk of early or late mortality as estimated by multivariable analysis among patients in the injectable group

Characteristic	Survived (n=500)	Early Death: died ≤3 months after treatment start (n=108)	Late Death: died >3 to 24 months after treatment start (n=91)	aRD per 100 (95%CI) Death Ever vs. Survival	aRD per 100 (95%CI) Early Death vs. Survival	aRD per 100 (95%CI) Late Death vs. Survival
Age, median (IQR), per 10y	33 (27 to 40)	39 (31 to 46)	35 (29 to 44)	6 (3 to 9)	4 (3 to 6)	2 (0 to 3)
Sex						
Female	228 (73.5%)	42 (13.5%)	40 (12.9%)	0 (reference)	0 (reference)	0 (reference)
Male	272 (69.9%)	66 (17%)	51 (13.1%)	1 (-4 to 7)	0 (-3 to 4)	-1 (-3 to 1)
AFB Smear at Baseline						
Negative	230 (71.4%)	42 (13%)	50 (15.5%)	0 (reference)	0 (reference)	0 (reference)
Positive	186 (75%)	40 (16.1%)	22 (8.9%)	-1 (-7 to 5)	2 (-2 to 5)	-4 (-9 to -0.3)
Unknown	84 (65.1%)	26 (20.2%)	19 (14.7%)	3 (-8 to 14)	2 (-5 to 9)	-1 (-8 to 5)
Culture at Baseline						
Negative	96 (76.2%)	15 (11.9%)	15 (11.9%)	0 (reference)	0 (reference)	0 (reference)
Positive	244 (73.1%)	48 (14.4%)	42 (12.6%)	8 (1 to 14)	3 (-1 to 6)	5 (1 to 9)
Unknown	160 (66.9%)	45 (18.8%)	34 (14.2%)	9 (1 to 18)	5 (-2 to 11)	6 (1 to 10)
HIV-Status						
Negative	179 (79.6%)	31 (13.8%)	15 (6.7%)	0 (reference)	0 (reference)	0 (reference)
Positive on ART	302 (68.6%)	67 (15.2%)	71 (16.1%)	8 (2 to 15)	2 (-2 to 5)	7 (2 to 11)
Positive not on ART	19 (55.9%)	10 (29.4%)	5 (14.7%)	22 (4 to 41)	11 (-4 to 26)	4 (-5 to 14)
Previous Treatment						
Never Treated	312 (75.5%)	63 (15.3%)	38 (9.2%)	0 (reference)	0 (reference)	0 (reference)
Treated with First Line Drugs	188 (65.7%)	45 (15.7%)	53 (18.5%)	8 (1 to 15)	0 (-3 to 3)	5 (-0.3 to 9)
Isoniazid Resistance						
Susceptible	177 (70.5%)	34 (13.5%)	40 (15.9%)	0 (reference)	0 (reference)	0 (reference)
Resistant	217 (76.4%)	37 (13%)	30 (10.6%)	-2 (-8 to 5)	-1 (-5 to 2)	-1 (-3 to 2)
Unknown	106 (64.6%)	37 (22.6%)	21 (12.8%)	7 (-1 to 15)	6 (-0.4 to 12)	0 (-2 to 3)

Abbreviations: AFB, acid fast bacilli; aRD, adjusted risk difference; 95% CI, 95% confidence interval; ART, antiretroviral treatment; IQR, interquartile range

*Notes: Comparisons were done only on patients who were alive during the analytical period (e.g., analyses of death >3 months vs. survival were only done on patients who were alive after 3 months of treatment). Percentages represent row percentages. Model is adjusted for all factors listed here, including province of treatment.

Appendix Table 9: Risk of early or late mortality as estimated by multivariable analysis among patients in the bedaquiline group

Characteristic	Survived (n=526)	Early Death: died ≤3 months after treatment start (n=63)	Late Death: died >3 to 24 months after treatment start (n=99)	aRD per 100 (95%CI) Death Ever vs. Survival	aRD per 100 (95%CI) Early Death vs. Survival	aRD per 100 (95%CI) Late Death vs. Survival
Age, median (IQR), per 10y	41 (33 to 51)	47 (36 to 59)	41 (33 to 54)	4 (2 to 6)	2 (1 to 3)	1 (-1 to 3)
Sex						
Female	203 (76.6%)	26 (9.8%)	36 (13.6%)	0 (reference)	0 (reference)	0 (reference)
Male	323 (76.4%)	37 (8.7%)	63 (14.9%)	0 (-5 to 4)	-1 (-2 to -0.1)	1 (-4 to 6)
AFB Smear at Baseline						
Negative	244 (79.5%)	26 (8.5%)	37 (12.1%)	0 (reference)	0 (reference)	0 (reference)
Positive	224 (75.4%)	25 (8.4%)	48 (16.2%)	3 (-3 to 8)	1 (-0.3 to 2)	3 (-3 to 9)
Unknown	58 (69%)	12 (14.3%)	14 (16.7%)	7 (-6 to 19)	2 (-0.1 to 4)	6 (-4 to 16)
Culture at Baseline						
Negative	108 (84.4%)	6 (4.7%)	14 (10.9%)	0 (reference)	0 (reference)	0 (reference)
Positive	263 (76.9%)	23 (6.7%)	56 (16.4%)	8 (2 to 14)	-2 (-3 to -1)	6 (-1 to 12)
Unknown	155 (71.1%)	34 (15.6%)	29 (13.3%)	10 (1 to 18)	4 (0.2 to 7)	1 (-6 to 8)
HIV-Status						
Negative	147 (75.4%)	20 (10.3%)	28 (14.4%)	0 (reference)	0 (reference)	0 (reference)
Positive on ART	372 (77.8%)	37 (7.7%)	69 (14.4%)	1 (-4 to 6)	0 (-1 to 1)	0 (-6 to 5)
Positive not on ART	7 (46.7%)	6 (40%)	2 (13.3%)	25 (-7 to 57)	6 (-23 to 35)	2 (-29 to 34)
Previous Treatment						
Never Treated	328 (79.2%)	36 (8.7%)	50 (12.1%)	0 (reference)	0 (reference)	0 (reference)
Treated with First Line Drugs	198 (72.3%)	27 (9.9%)	49 (17.9%)	5 (0.4 to 10)	-1 (-1 to 0.1)	6 (1 to 12)
Isoniazid Resistance						
Susceptible	200 (74.9%)	28 (10.5%)	39 (14.6%)	0 (reference)	0 (reference)	0 (reference)
Resistant	213 (79.2%)	18 (6.7%)	38 (14.1%)	-4 (-9 to 2)	0 (-1 to 1)	-2 (-8 to 3)
Unknown	113 (74.3%)	17 (11.2%)	22 (14.5%)	2 (-7 to 11)	-1 (-3 to 0.2)	3 (-4 to 11)

Abbreviations: AFB, acid fast bacilli; aRD, adjusted risk difference; 95% CI, 95% confidence interval; ART, antiretroviral treatment; IQR, interquartile range

*Notes: Comparisons were done only on patients who were alive during the analytical period (e.g., analyses of death >3 months vs. survival were only done on patients who were alive after 3 months of treatment). Percentages represent row percentages. Model is adjusted for all factors listed here, including province of treatment.
